# Socio-demographic factors impact disabilities caused by perinatal asphyxia among Chinese children

**DOI:** 10.1371/journal.pone.0248154

**Published:** 2021-03-05

**Authors:** Deng Ao, Shuai Guo, Chunfeng Yun, Xiaoying Zheng

**Affiliations:** 1 Institute of Population Research/WHO Collaborating Center on Reproductive Health and Population Science, Peking University, Beijing, China; 2 Department of Preschool Education, Teacher’s College of Beijing Union University, Beijing, China; 3 The MOH Key Laboratory of Geriatrics, Beijing Hospital, National Center of Gerontology, Beijing, China; University of Western Australia, AUSTRALIA

## Abstract

**Background:**

Disabilities caused by perinatal asphyxia will burden child health and well-being. To date, our understanding about the situation and risk factors of perinatal asphyxia-induced disabilities among Chinese children is still limited.

**Objectives:**

To evaluate the prevalence and socio-demographic risk factors of disabilities caused by perinatal asphyxia among Chinese children in 2006 and compare disability trajectories across different socio-demographic status.

**Methods:**

Cross-sectional data came from the 2006 China National Survey on Disability which includes a total of 616,940 children aged 0–17 years old was employed in the investigation. Perinatal asphyxia-induced disabilities were identified by following the guidance in consensus manuals. Population-weighted numbers and prevalence rates were investigated, and multivariable logistic regression was performed to evaluate associations between disabilities and socio-demographic factors. Adjusted predictions at representative values were computed to compare the disability trajectories relative to significant socio-demographic variables.

**Results:**

The prevalence rate of disabilities caused by perinatal asphyxia was 7.70 per 10,000 children (95% CI: 7.01–8.39). Male (OR 1.81, 95% CI: 1.47–2.23) and low family income (OR: 1.73, 95% CI: 1.21–2.49) have higher and the increase of per additional year of age (OR: 0.89, 95% CI: 0.88–0.91) has lower probability of being disabilities caused by perinatal asphyxia. Further disability trajectories showed that differences in probability between gender and family income group were more evident before age 7 and weakened with increasing age.

**Conclusions:**

Our results showed that both demographic and socioeconomic characteristics are risk factors for disabilities caused by perinatal asphyxia. Of these, gender and family income have much higher impact than other factors on the prevalence rate of disabilities caused by perinatal asphyxia at infants and young children. Multiple society sectors should increase their effort to bring about fundamental social change to prevent disabilities caused by perinatal asphyxia, especially concerning younger children and their families.

## Introduction

Perinatal asphyxia is clinically defined by the World Health Organization (WHO) as a newborn failing “to initiate or maintain regular breathing at birth” [1, 2]. The incidence of perinatal asphyxia is 1–8 per 1,000 live births, depending on the definition and study population [3]. In China, a previous study estimated that the annual incidence in 2010 was 50 per 1,000 live births [4]. Severe perinatal asphyxia can cause nerve damage and the malfunction of organs, muscles, and tissues [5], or even result in neonatal death or long-term disabilities [6]. In China, in 2006, 2.06 per 10,000 live-birth infants died because of perinatal asphyxia [7]. Children who have survived may have lifelong disability. For instance, birth asphyxia and birth trauma account for 8.6% of occurrences of hearing disabilities [4], and 6.3% of intellectual disabilities among the children at 0–6 years old in China [8]. Disabilities in children can entail a massive burden on individuals, families, and societies [9]. In cases of perinatal asphyxia causing functional impairment to children, comprehensive care and rehabilitation services should be provided to increase their quality of life and help them integrate into society [10]. The recognition of risk factors may provide prior knowledge for appropriate early intervention and rehabilitation training. However, to date, our understanding about the prevalence rate and relative risk factors of perinatal asphyxia-induced disabilities among children in the general Chinese population is still very limited.

The social landscape of a country, including its culture and character of poverty among its citizens, impacts the form and magnitude of disability risks [11]. In the late 1970s, China embarked on economic reforms and implemented the One-Child Policy. The new sociopolitical conditions resulting from these new policies influenced people’s socio-demographic profile and the households to which they belong intricately and uncertainly [12]. Thus, understanding the impact of socio-demographic factors on the risk of perinatal asphyxia-induced disabilities among children aged 0–17 years in China is essential for improving the population’s health and lives. However, inadequate information about this issue makes it hard to be attempted. Moreover, how the influence changes with age among children remains unclear.

To this end, we attempt to analyze the prevalence of disabilities caused by perinatal asphyxia and their socio-demographic factors among Chinese children, by using data collected from the 2006 China National Survey on Disability (CNSD), a nationally representative population-based survey. We also assessed socio-demographic differences in disability trajectories from 0 to 17 years old. Our findings will enhance the understanding of disabilities caused by perinatal asphyxia and inform population health policies to reduce their risks in similar settings.

## Methods

### Study population

The present study examined a subpopulation of children with disabilities caused by perinatal asphyxia from the 2006 CNSD, which was conducted from 1 April to 31 May 2006. As cross-sectional data, the detailed sampling procedure of CNSD has already been described in detail [13–15]. This national survey used a multistage, stratified random cluster sampling design, with probability proportional to size. It covered all 31 province-level administrative regions (provinces, autonomous regions, and municipalities directly under mainland China’s central government). In each region, sampling strata were defined based on population, geographic and economic data. A four-level sampling frame was applied within each stratum to comprise four administrative units (county, town, village, and community). A total of 734 counties were sampled at the first level; then, four towns in each county, two villages in each town, and one community in each village were sampled. Finally, 5964 communities were sampled in 2006 [16]. The final sample size of the survey was 2,526,145, representing 1.9 per 1,000 non-institutionalized inhabitants of China [9].

The prevalence and causes of different types of disabilities, the family characteristics of people with disabilities, and the needs of this population for social services and support were investigated—the study adhered to the Declaration of Helsinki principles. The surveys were approved by The State Council of China (Guo Ban Fa No. 73 [2004]). All respondents provided consent to participate in the survey and clinical diagnosis. The data were anonymized before analysis. In this study, we only considered survey respondents aged 0–17 years old, born between 1 April 1988 and 31 May 2006.

### Measures

More than 20,000 interviewers, 6,000 physicians with various specializations, and 5,000 survey assistants participated in this survey. Pre-survey information such as the number of households, population size, and persons with disabilities in the sampling community was collected before 25 March 2006 [15]. During the formal household survey, every family member of the selected households was interviewed for basic information, socio-demographic information about the households was self-reported by the householder. Then, trained field interviewers conducted a screen scale of disabilities for every family member of the household aged 7 years and above. All those suspected of having disabilities and all children aged 0–6 years received medical examinations performed by different designated physicians to accurately diagnose the disability and confirm its primary causes [17, 18].

Perinatal asphyxia-induced disabilities were defined as hearing and/or intellectual disabilities that resulted from perinatal asphyxia, as determined by an expert panel consensus. Perinatal asphyxia was described as a failure to start regular respiration within 1 min of birth, as diagnosed according to a 1 min Apgar score ≤ 7 [19]. The definition and classification of hearing and intellectual disabilities were established by the Expert Committee of the 2006 CNSD, based on the WHO International Classification of Functioning, Disability, and Health (WHO-ICF) [20].

Hearing disabilities are referred to permanent hearing impairment of varying degrees from any cause or the inability to hear at all or to hear nearby sounds or vocal expressions in a way that negatively impacts the person’s daily life and participation in social activities [9]. Pure Tone audiometry was performed for all children over 3 years old; for children aged 0–3 years, pure tones were replaced by warble tones. Children were diagnosed as having a hearing disability if their averaged hearing threshold of the better ear was over 60 dB HL (children aged 0–3 years old) or over 40 dB HL (children aged 4–17 years old) [21].

Intellectual disabilities were defined concerning a development quotient < 75 (children aged 0–6 years old) measured by Gesell Development Inventory or intelligence quotient < 70 (children aged 7–17 years old) assessed by Wechsler Intelligence Scale for Children-Chinses Revision, and classified as a cognitive disability, manifesting in cognitive, affective, or behavioral disorders that limited the person’s daily life and restricted social participation [21, 22]. Home re-visits were performed for post-survey quality checks after the field investigations were conducted.

### Study variable definition

Perinatal asphyxia-induced disability was categorized as binary, i.e., yes or no. We further categorized age groups (0–3, 4–6, or 7–17 years old), neonate gender (male or female), residence (rural areas or urban areas), family size (≤ 3 persons or larger), and regions (west, central east). As socio-demographic information of the household, annual family income was self-reported by the householder according to the sum of all the family members’ economic income, including wages, net business income, property income, and transfer income. The annual family income per capita was calculated by dividing annual family income by the number of household people. It was categorized as binary regarding the national average annual family income, i.e., ≤ national average or higher. In 2006, the national average annual family income was 11,759 Ren min bi (RMB) for urban areas and 3,578 RMB for rural areas.

### Statistical analyses

We used standard weighting procedures calculating the inverse probability of inclusion for individual survey respondents in the multistage sampling frame to construct sample weights, taking into account the complex survey sample design [23]. First, weighted numbers and prevalence of disability were estimated, with 95% confidence intervals (CIs), for the overall population and different population segments. Chi-square tests were performed to examine the differences in prevalence between socio-demographic variables. Second, multivariable logistic regression was used to calculate the adjusted odds ratios (ORs) and 95% CI. We estimated disability as a linear function of age after checking for the linear association of age with perinatal asphyxia-induced disabilities. (see S1 Table) Finally, adjusted predictions were estimated using adjusted predictions at representative values for comparing disability trajectories regarding significant socio-demographic characteristics. The survey data analyses in STATA version 14.1 were used to perform all the data analyses. Two-sided P values < 0.05 were considered to indicate statistical significance.

## Results

### Prevalence of children with disabilities caused by perinatal asphyxia

Among the 616,940 (weighted at 324,772,737) children investigated in this survey, a total of 445 cases of disability (weighted at 250,065) were caused by perinatal asphyxia. Moreover, 9 cases (2.0%) were hearing disabilities, 414 (93.0%) were intellectual disabilities, and about 5.0% of the total cases (22) were both hearing and intellectual disabilities. The weighted prevalence rate was 7.7 per 10,000 (95% CI: 7.0–8.4). As shown in Table 1, the weighted prevalence rate of disabilities caused by perinatal asphyxia was significantly higher among males, children aged 4–6 years old, and children with an average annual family income equal to or lower than the national average. There were no significant differences in the prevalence of disabilities in children for socio-demographic variables like residence, family size, and region.

**Table 1:** Prevalence of disabilities caused by perinatal asphyxia among Chinese children.

	Sample N	Weighted N (%)	Disabilities caused by perinatal asphyxia			*P*^a^
			Sample N	Weighted N	Weighted prevalence per 10000 (95% CI)	
Total	616940	324772737 (100.0)	445	250065	7.7 (7.0–8.4)	
Age group						
0–3	109496	57977238 (17.9)	145	83597	14.4 (12.2–16.7)	<0.001
4–6	82497	43394803 (13.4)	123	71658	16.5 (13.7–19.3)	
7–17	424947	233400695 (71.9)	177	94810	4.2 (3.6–4.9)	
Neonate gender						
Male	330053	174458165 (53.7)	305	170631	9.8 (8.7–10.9)	<0.001
Female	286887	150314572 (46.3)	140	79434	5.3 (4.4–6.1)	
Residence						
Rural	449500	241366262 (74.3)	326	188186	7.8 (7.0–8.6)	0.659
Urban	167440	83406474 (25.7)	119	61879	7.4 (6.1–8.7)	
Annual family income per capita						
≤ National average	513431	274200919 (84.4)	387	220821	8.1 (7.3–8.8)	0.028
> National average	103509	50571817 (15.6)	58	29244	5.8 (4.3–7.3)	
Family size						
> 3 people	432505	229009694 (70.5)	302	170329	7.4 (6.6–8.3)	0.285
≤ 3 people	184435	95763042 (29.5)	143	79736	8.3 (7.0–9.6)	
Region						
West	221162	100548819 (31.0)	153	76317	7.6 (6.4–8.7)	0.970
Central	207031	115421033 (35.5)	150	90195	7.8 (6.6–9.0)	
East	188747	108802884 (33.5)	142	83553	7.7 (6.4–8.9)	

^a^ Chi-square test for differences of categorical variables.

### Risk factors for child disabilities caused by perinatal asphyxia

As shown in Table 2, the risk of disabilities caused by perinatal asphyxia was associated with the male gender (OR: 1.81, 95%: CI 1.47–2.23) and low average annual family income (OR: 1.73, 95% CI: 1.21–2.49). Age increase was a significant protective factor (OR: 0.89, 95% CI: 0.88–0.91).

**Table 2:** Factors associated with disabilities caused by perinatal asphyxia among Chinese children.

	OR (95% CI)	*P*
Per additional year of age	0.89 (0.88–0.91)	< 0.001
Neonate gender		
Male	1.81 (1.47–2.23)	< 0.001
Female	1.00 (reference)	
Residence		
Rural	0.81 (0.62–1.07)	0.135
Urban	1.00 (reference)	
Annual family income per capita		
≤national average	1.76 (1.22–2.53)	0.003
>national average	1.00 (reference)	
Family size		
>3 people	0.82 (0.66–1.02)	0.079
≤3 people	1.00 (reference)	
Region		
West	0.93 (0.73–1.18)	0.545
Central	0.93 (0.74–1.19)	0.583
East	1.00 (reference)	

### Disability trajectories relative to significant socio-demographic variables

**Fig 1:**
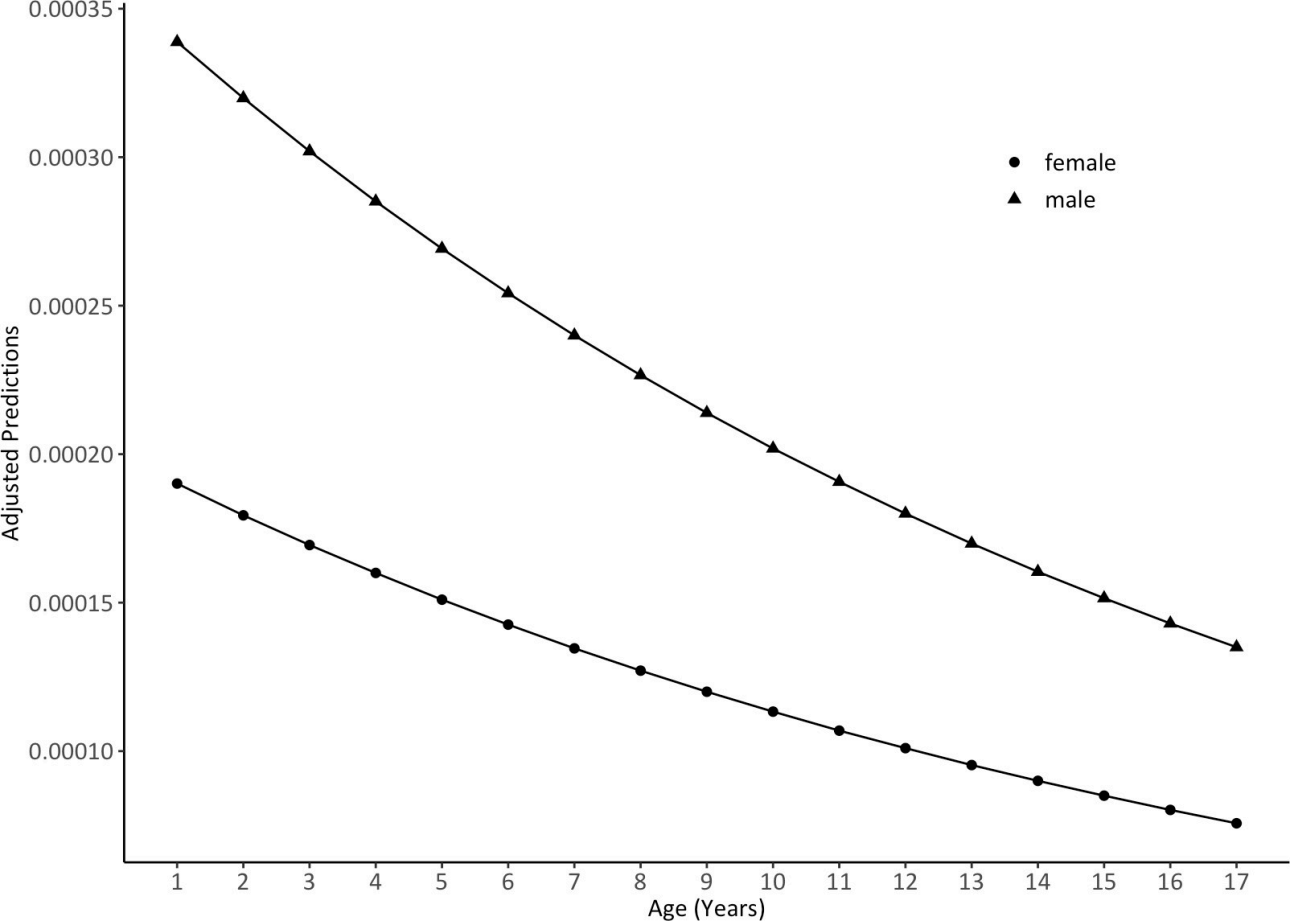
Predicted trajectories for the disabilities caused by perinatal asphyxia, by sex.

**Fig 2:**
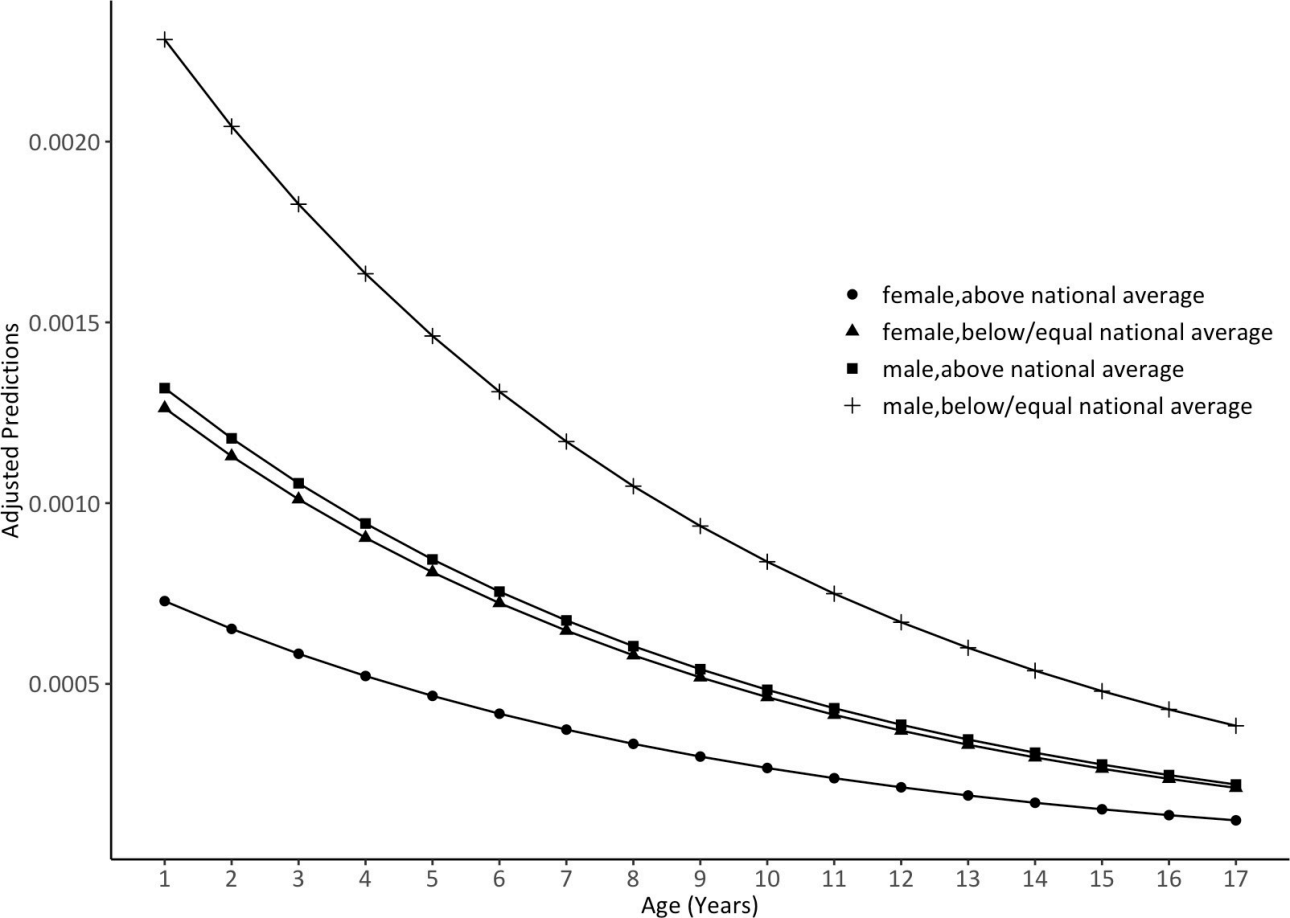
Predicted trajectories for the disabilities caused by perinatal asphyxia, by sex and family income groups.

As shown in Fig 1, the probability of disabilities caused by perinatal asphyxia decreased with age. However, boys had a higher probability in lower age groups, as the trend of their probability decreased faster than that of girls. Overall, the probability gap between boys and girls was narrowed with age increase. Fig 2 depicts the effects of income on the probability of disabilities caused by perinatal asphyxia between gender groups. The low-family-income group has significantly higher probability of disabilities caused by perinatal asphyxia than the high-family-income group, but its probability curve in this group decreased more over the 17 years of age. The phenomena is more significant among boys when comparing to girls, which have resulted in the most significant advantage for the low-family-income boys. Moreover, low-family-income girls have similar disability trajectories to that of higher-family-income boys, especially when they approached 17.

## Discussion

Our data, drawn from a national-wide representative survey, indicate that approximately 8 in every 10,000 children in China have suffered from hearing and/or intellectual disabilities caused by perinatal asphyxia. As a result of general socioeconomic developments and reforms in the health sector, maternal care utilization, including prenatal care and delivery care, have been significantly improved, and neonatal mortality have trended to decrease year over year since 1991 in China. [24–26]. In parallel, with the improvements in prenatal diagnosis and obstetric care have been brought to bear, perinatal asphyxia by incidence has been declined steadily [27]. Although the incidence of perinatal asphyxia was reduced from 6.3% to 2.9% in 2003–2008 [28], the neonatal mortality rate caused by perinatal asphyxia declined much faster, which is 8.2% per year on average between 1996 and 2008 [29]. This indicates that neonatal survival improvement may increase the proportion of children surviving perinatal asphyxia, which is in agree with the point of improving child well-being to preventing disabilities.

Our results indicate that the prevalence of disabilities depends on substantially according to demographic and socioeconomic characteristics. In particular, younger children, boys, and children from low-income families all exhibited a significantly higher prevalence of perinatal asphyxia-induced disabilities. Besides, disparities in disabilities caused by perinatal asphyxia between boys and girls narrowed with age.

We also found that the national prevalence rate of younger children, especially children aged 4–6 years old, born between 2000 and 2002, was higher than 7–17 years old children. We speculated that the phenome could be partly explained by the decline of postnatal care [30]. Developmental screening tests should be administered at the 9-, 18-, and 30-month visits [31]. However, the proportion of women receiving at least one postnatal care visit dropped slightly, from 56.5% in 1991–1993 to 54.1% in 2001–2003 [32]. Missing the optimal time for detection and treatment could result in developmental problems or disabilities caused by perinatal asphyxia. Meanwhile, some experts consider that this may be a result of uneven development in children [8]. Although the hearing disabilities would be less likely to decrease with age because many of them are defined as permanent disabilities, some intellectual disabilities may go away as children are treated and get older. What’s more, the survival of disabled persons over time will also determine the disability prevalence [33]. However, this explanation is speculative. Further study should be conducted to determine the decline in disabilities by age. As indicated in Fig 1, disability probability curves for both sexes were at high levels in the younger group and decreased with age to a low level, which indicates that there is still room for improvement in reducing risk among infants and young children while also leading to the conclusion that current levels of care in early diagnosis, screening, and rehabilitation are far from sufficient.

Our statistical analyses showed that boys were at higher risk than girls. Son preferences and the One-Child Policy may explain this phenomenon. In China, parents have long favored sons over daughters [31]. With restrictions on the number of births under the One-Child Policy, most mothers are likely to have at least one surviving son [34, 35]. Thus, boys may survive regardless of whether they are disabled, but girls with defects may suffer from abandonment at birth, possibly even infanticide [36, 37]. As shown in Fig 2, boys’ disability trajectories were higher than those of girls, regardless of the family income level, suggesting that son preferences may have led to higher survival rates for boys who experienced perinatal asphyxia or even disability. Considering the change of probabilities with age, we can speculate that even parents in poverty may be more likely to find rehabilitation assistance for boys with disabilities [36, 38].

Low family income was another risk factor for disabilities caused by perinatal asphyxia. Low-income families may encounter many difficulties, including fewer resources and lower levels of educational attainment [39]. Early intervention was previously less common and not nearly sufficient for younger children who need it [30, 40]. So, they are more likely to reduce health-care-seeking behavior and believe in karma. By contrast, higher-income parents may have the ability to pay for services and make a tremendous effort to seek services [30].

Socio-economic disparities between regions are not reflected in the prevalence of prenatal asphyxia-induced disabilities in the present study. This phenomenon may be due to the following two reasons. Firstly, though the difference in per capita GDP at the provincial level is significant [41], in 2006, per capita GDP between central and western regions were close (12844 yuan vs. 10932 yuan) [42], which indicated the similar social and economic development in these two regions. Secondly, the China Neonatal Resuscitation Program (NRP), aiming to reduce prenatal asphyxia and related mortality among 20 provinces with high neonatal mortality, was implemented in 2004. The target provinces contained 92% of the western provinces and 75% provinces from the central region, only 3 from 11 eastern provinces were selected. As the evaluation study showed that China NRP has dramatically reduced the prenatal asphyxia incidence and mortality from asphyxia in the target provinces from 2003 to 2008 [43]. The impact of the program may narrow down the gap in differences in disabilities caused by perinatal asphyxia between eastern and the other two regions.

The most interesting finding of this study is that the probability gap between the higher and low-family-income groups in disability trajectories was more significant among boys than girls. One possible explanation for this could be the differential response to the One-Child Policy between income groups. As mentioned above, One-Child Policy and son preferences would result in more surviving boys regardless of disability. Moreover, under this policy, parents who exceeded their fertility limits would be forced to pay fines and penalties. In response to this situation, women who gave birth to a second, third, and following child were less likely to use prenatal care or professional delivery assistance to evade financial penalties [32, 44]. Families with higher annual incomes, however, would rather pay fines and receive prenatal care to ensure the younger children were maintained in good health. By contrast, low-income families could not invest as much to ensure care for their first child and may even have chosen not to use the standard medical service at all for the second and following child in the family due to the fines. Thus, the policy brought further disadvantages to boys regarding access to medical services from low-income families. However, this explanation is speculative. Further studies should examine it in more detail.

Our findings have important implications for prevention and rehabilitation policy. The public and private sectors should make a greater effort to reduce gender inequality. Strategies to address cultural norms should be incorporated into such interventions. China has experienced an ongoing decline in its sex ratio at birth since 2010, reaching a ratio of 113.55 male-to-female births in 2015 [45]. However, the demand for simple labor (distinguishing from skilled labor) and the preference for sons continue to exist [38, 46–48]. Gender inequality continues to be a significant problem in China. This study also found that targeting low-income families for secondary prevention and rehabilitation may decrease the adverse effects of perinatal asphyxia. This is especially important to reduce disparities in access to comprehensive care [49]. As resources become more available and payment structures for delivery services improve, families tend to seek care for both sons and daughters to ensure that both have a better chance at a normal life experience. The underlying dynamics of poverty are changing as the economy develop rapidly. A dramatic fall has been seen in the number of people living below the poverty line in China, whether measured by the income poverty line established by the Chinese government or international levels of absolute poverty [49]. However, the disparities in maternal health and the utilization of rehabilitation services between low- and high-income households continue to exist [44]. As gender and income disparities for disabilities due to perinatal asphyxia decline with age, early intervention should be provided to improve children’s development and help them have a normal life experience well before they reach 7 years of age.

This study has provided a broad understanding of the prevalence of disabilities caused by perinatal asphyxia and its relationship with various vital socioeconomic factors in China for the first time. However, the present study also had some weaknesses. Our reliance on data from a cross-sectional survey may prevent us from making causal inferences. There is a strong need to conduct longitudinal studies to determine causality. The lack of individual-level data on certain variables prevented us from examining possible mediators (e.g., information of the antenatal care and health care utilization) to better understand the mechanisms underlying these associations. Besides, potential higher mortality among the younger children with disabilities caused by perinatal asphyxia may result in underestimation of the disability prevalence in this group and further lead to bias in the association between disability and age. We cannot verify this effect because the current data did not have information on child deaths. Future research should consider collecting relevant data to provide a complete picture. Finally, the data of this study is outdated (14 years old). Patterns may look very different in recent years due to the recent Two-Child Policy instituted in 2015. This should be a future research area.

## Conclusions

In 2006, more than 250,000 children with disabilities were caused by perinatal asphyxia in China. Adverse demographic and socioeconomic characteristics may be risk factors for disabilities caused by perinatal asphyxia. Gender inequality and disparities in utilization of maternal health and rehabilitation services between low- and higher-income households continue to exist. For younger children, gender and income inequalities are even more severe. Multiple society sectors should make a more significant effort to bring about fundamental social change and prevent the occurrence of disabilities caused by perinatal asphyxia.

## Supporting information

S1 Table Factors associated with disabilities caused by perinatal asphyxia among Chinese children (with age-squared in the regression model). (DOCX)
