# Supplementary material for: Socio-demographic factors impact disabilities caused by perinatal asphyxia among Chinese children
Source: PLoS One. 2021 Mar 5;16(3):e0248154. doi: 10.1371/journal.pone.0248154 (PMC7935314; doi:10.1371/journal.pone.0248154)
Supplement: S1 Table — (DOCX) [file pone.0248154.s001.docx]

**S1 Table. Factors associated with disabilities caused by perinatal asphyxia among Chinese children (with age-squared in the regression model)**

|  | **OR (95% CI)** | ***P*** |
| --- | --- | --- |
| Per additional year of age | 0.95 (0.89-1.01) | 0.095 |
| Per additional year of age (squared) | 1.00 (0.99-1.00) | 0.104 |
| Neonate gender |  |  |
| Female | 1.00 (reference) |  |
| Male | 1.81(1.47-2.23) | <0.001 |
| Residence |  |  |
| Urban | 1.00 (reference) |  |
| Rural | 0.81(0.62-1.06) | 0.131 |
| Annual family income per capita |  |  |
| >national average | 1.00 (reference) |  |
| ≤national average | 1.76(1.22-2.54) | 0.002 |
| Family size |  |  |
| ≤3 people | 1.00 (reference) |  |
| >3 people | 0.84(0.67-1.04) | 0.103 |
| Region |  |  |
| West | 0.93(0.73-1.18) | 0.565 |
| Central | 0.93(0.73-1.18) | 0.559 |
| East | 1.00 (reference) |  |

As we can see that the ORs of continuous age and age squared are close to 1 and neither one is significant. On reflection, we chose to only keep continous age in the current model.
